# Aerosol-induced atmospheric heating rate decreases over South and East Asia as a result of changing content and composition

**DOI:** 10.1038/s41598-020-76936-z

**Published:** 2020-11-18

**Authors:** S. Ramachandran, Maheswar Rupakheti, Mark G. Lawrence

**Affiliations:** 1 Physical Research Laboratory, Ahmedabad, India; 2 Institute for Advanced Sustainability Studies, Potsdam, Germany; 3 Institute for Environmental Sciences and Geography, University of Potsdam, Potsdam, Germany

**Keywords:** Atmospheric science, Climate change

## Abstract

Aerosol emissions from human activities are extensive and changing rapidly over Asia. Model simulations and satellite observations indicate a dipole pattern in aerosol emissions and loading between South Asia and East Asia, two of the most heavily polluted regions of the world. We examine the previously unexplored diverging trends in the existing dipole pattern of aerosols between East and South Asia using the high quality, two-decade long ground-based time series of observations of aerosol properties from the Aerosol Robotic Network (AERONET), from satellites (Moderate Resolution Imaging Spectroradiometer (MODIS) and Ozone Monitoring Instrument (OMI)), and from model simulations (Modern-Era Retrospective Analysis for Research and Applications, version 2 (MERRA-2). The data cover the period since 2001 for Kanpur (South Asia) and Beijing (East Asia), two locations taken as being broadly representative of the respective regions. Since 2010 a dipole in aerosol optical depth (AOD) is maintained, but the trend is reversed—the decrease in AOD over Beijing (East Asia) is rapid since 2010, being 17% less in current decade compared to first decade of twenty-first century, while the AOD over South Asia increased by 12% during the same period. Furthermore, we find that the aerosol composition is also changing over time. The single scattering albedo (SSA), a measure of aerosol’s absorption capacity and related to aerosol composition, is slightly higher over Beijing than Kanpur, and has increased from 0.91 in 2002 to 0.93 in 2017 over Beijing and from 0.89 to 0.92 during the same period over Kanpur, confirming that aerosols in this region have on an average become more scattering in nature. These changes have led to a notable decrease in aerosol-induced atmospheric heating rate (HR) over both regions between the two decades, decreasing considerably more over East Asia (− 31%) than over South Asia (− 9%). The annual mean HR is lower now, it is still large (≥ 0.6 K per day), which has significant climate implications. The seasonal trends in AOD, SSA and HR are more pronounced than their respective annual trends over both regions. The seasonal trends are caused mainly by the increase/decrease in anthropogenic aerosol emissions (sulfate, black carbon and organic carbon) while the natural aerosols (dust and sea salt) did not change significantly over South and East Asia during the last two decades. The MERRA-2 model is able to simulate the observed trends in AODs well but not the magnitude, while it also did not simulate the SSA values or trends well. These robust findings based on observations of key aerosol parameters and previously unrecognized diverging trends over South and East Asia need to be accounted for in current state-of-the-art climate models to ensure accurate quantification of the complex and evolving impact of aerosols on the regional climate over Asia.

## Introduction

Anthropogenic aerosol emissions over Asia are changing^1,2^. This region is extremely sensitive to climate change owing to a dependency on the strong seasonal variation in monsoon and the very high population densities, including a large poor populace^1^. The associated risks involve freshwater availability, climate extremes and large societal costs^1^. Thus understanding and quantifying the climate impacts of Asian aerosols from local to regional to global scales is crucial^3,4^. Model simulations and satellite observations show that the geographical distributions of emissions of trace gases and aerosol particles that can alter the atmospheric energy balance have changed in the last decade due to economic growth and air pollution regulations^2^. This is especially the case over Asia, where a dipole in aerosol pollution has been noted between South and East Asia^1^, with a qualitative difference in the trend of emissions: over South Asia, the emissions have been increasing, while over East Asia they have been decreasing. The climate over Asia is governed by a variety of land–ocean-atmospheric processes, varying across spatial and temporal scales, making it a challenging task to simulate and predict the climate implications of such emerging patterns of aerosol forcing^1^.

**Figure 1:**
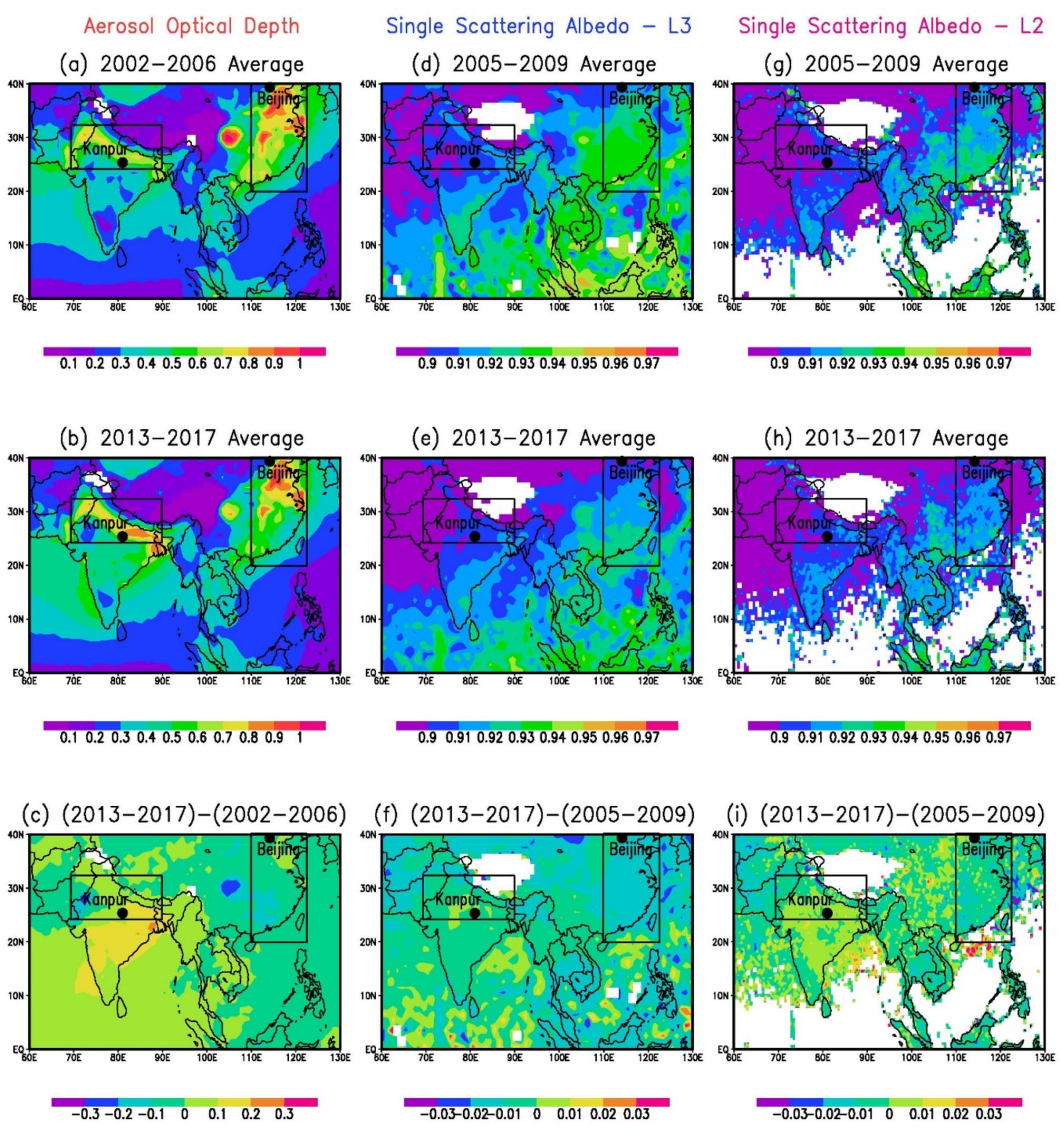
Changing aerosol patterns over South Asia and East Asia: MODIS Terra version 6.1 level-3 monthly AOD at a wavelength of 0.55 µm averaged for (**a**) 2002–2006 and (**b**) 2013–2017. OMI OMAERUV v003 level-3 SSA at a wavelength of 0.388 µm averaged for (**d**) 2005–2009 and (**e**) 2013–2017, and OMI OMAERO v003 level-2 SSA at a wavelength of 0.388 µm averaged for (**g**) 2005–2009 and (**h**) 2013–2017. The spatial differences in AOD and SSA between the two periods are shown in (**c**), (**f**), and (**i**) respectively. The AOD and SSA (level-3) data were downloaded from https://giovanni.gsfc.nasa.gov/giovanni/. SSA (level-2) data were downloaded from https://disc.gsfc.nasa.gov/datasets/OMAERO_003/summary.

Atmospheric aerosols contribute to climate change by both heating and cooling the atmosphere, and cooling the surface. The net global radiative effect due to aerosol heating and cooling is negative and has offset a substantial portion of the warming due to greenhouse gases^5^. However, there exists at least a factor of three uncertainty in the aerosol radiative forcing estimates^5^. One of the main factors causing this is the uncertainty in the atmospheric solar heating by black carbon (BC). Models grossly underestimate aerosol absorption in many regions, especially over Asia^6^. East Asia, in particular the North China Plain (NCP), and South Asia, notably the Indo-Gangetic Plain (IGP) are global air pollution hotspots. Previous studies on aerosol properties and emissions over Asia including the IGP and the NCP regions were based on (i) in-situ data at one or only a few locations for a limited time period or only a select set of aerosol parameters^7–10^, (ii) inventories of aerosol emissions^11^, (iii) satellite data on AOD for a limited period, e.g., between 2000 and 2009^12^, and SO_2_ emissions between 2005 and 2015^13^, and (iv) multi-model simulations of radiative forcing estimates^14^. Recently, spatial distribution of microphysical and optical properties of aerosols and radiative forcing using the China Aerosol Remote Sensing Network was reported^15^. The climate impacts due to aerosols, including the impacts on the hydrological cycle and the Asian monsoon have been analyzed based on model simulated aerosol characteristics and observed trends^3,16^. However, as mentioned earlier, models grossly underestimate aerosol absorption in many regions^6,17^. Thus, the trends in aerosol impact on climate and climate change including the changes in precipitation and hydrological cycle based on model simulated aerosol characteristics over these regions may not be very accurate given the uncertainties and limitations of models and satellite data, and the lack of observation-based trend analysis. A complete, comprehensive and a more accurate analysis of aerosol characteristics, aerosol radiative effects and their trends over this region is crucial to develop, since the radiative forcing that results from the changing distribution patterns of aerosols is expected to be different from those observed in the late twentieth century^1^. These changes may trigger large scale atmospheric responses which will have wide-ranging impacts on climate, clouds, chemistry and other atmospheric processes extending well beyond source regions^1,18^. Thus, in order to understand the trends in aerosol properties over two important regions, East Asia and South Asia, we conduct the first analysis of high quality time series observations over a period of two-decades of the key climate-relevant aerosol parameters over Kanpur in South Asia and Beijing in East Asia, along with satellite observations that give a larger regional perspective (Fig. 1).

## Methods

### Satellite observations over Asia

We have utilized the MODIS Terra version 6.1 monthly level-3 aerosol optical depth (AOD) data at 0.55 µm, OMI OMAERUV v003 level-2 and level-3 single scattering albedo (SSA) data at 0.388 µm (https://giovanni.gsfc.nasa.gov/giovanni/). The uncertainty in level-2 (10 km) MODIS Terra version 6.1 AOD retrieval is ± 0.05 ± 0.15AOD^19^. The monthly level-3 AOD data is likely to have undergone some level of averaging between overestimation (positive) and underestimation (negative) errors. The MODIS Terra version 6.1 data, i.e., AOD retrieved using the Combined Dark Target and Deep Blue algorithms for land and ocean, and the AOD retrieved using the Dark Target algorithm for land are used^19^. Both the MODIS datasets are used as the Dark Target product of MODIS tends to discard a significant fraction of pixels with heavy aerosol loads leading to underestimation of the total aerosol scene^19^. The OMI SSA at 0.388 µm is used because retrievals at 0.50 µm in the OMAERUV dataset are not directly derived from the measurements, but converted from 0.388 µm retrievals assuming a spectral dependence model depending upon the chosen aerosol model. Due to some inherent uncertainties in the assumed spectral dependence, which might not represent all aerosol types/conditions globally, it is recommended to use the 0.388 µm SSA dataset^20^. The OMI observations have been affected by a possible external obstruction that perturbs both the measured solar flux and Earth radiance since mid-2007. This obstruction, affecting the quality of radiance at all wavelengths for a particular viewing direction, is referred to as a “row anomaly” since the viewing geometry is associated with the row numbers on the charge-coupled device detectors^20^. The row anomaly issue was detected first time in mid-2007 for a few rows which, over the period of operation, expanded to other rows in 2008 and later. At present, about half of the total 60 rows (viewing positions) across the track are identified and flagged as row anomaly affected positions for which no physical retrievals are being performed. Due to the row anomaly issue and some scan-related bias in the OMI SSA retrievals^20^, it is possible that the trends derived from the Level 3 OMAERUV SSA product might be affected and have some artefacts caused by this problem. Hence, we have also utilised the level-2 OMAERUV product for SSA derived using only the first 23 rows that are unaffected by the row anomaly throughout the OMI operation for comparison. SSA derived using viewing positions 1–30, and 31–60 were found to agree over regions dominated by carbonaceous or sulfate particles^20^. A global comparison of OMI retrieved SSA with that of co-located AERONET SSA found that 69% of OMI-AERONET SSA matchups agree within the absolute difference of ± 0.05 for all aerosol types^9^. Year-round OMI OMAERUV v003 level-3 and level-2 SSA data are available starting only from 2005.

A 5-year annual average AOD (Fig. 1a,b) and SSA (d, e, g, h) are plotted to avoid any differences/features arising out of inter-annual variability (it may be noted that changes in averages of 2002–2009 and 2010–2017 were not distinctly visible as they were smaller). Furthermore, two AERONET sites—Kanpur (26.5° N, 80.2° E, 123 m above mean sea level (asl)) in South Asia and Beijing (39.9° N, 116.4° E, 92 m asl) in East Asia are marked in the Figure. Kanpur, ~ 500-km downwind of the megacity New Delhi, located in the Indo-Gangetic Plain (IGP), is a densely populated, industrialized and heavily polluted location, and Beijing is a megacity located in the heavily industrialized and highly polluted North China Plain (NCP). More details on the ground-based in situ columnar aerosol data measured over Kanpur and Beijing are given in the next section. The general setting of the sites, and aerosol characteristics are very similar over Beijing and Kanpur; both are urban cities, and mainly emissions from fossil fuel combustion and biomass burning during all seasons, along with desert dust events that occur primarily in the spring season (March–May) contribute to the heavy aerosol pollution^21^. Both sites are heavily affected by regional emissions, in addition to their own local sources. The IGP and the NCP are influenced by the Asian summer monsoonal dynamics with northeasterly and southwesterly winds during the winter and summer monsoon, respectively. The aerosol types (emissions from fossil fuel and biomass burning, and dust) across India, in general, and over the IGP in particular were found to be the same throughout the year^7,17^, however, the percentage contribution of aerosol types were found to exhibit spatial and seasonal variations^17^. Similarly, over China and Beijing the spatial distribution of aerosol types was found to be the same whereas the percentage abundances of aerosol types varied as a function of settings (urban, rural and coastal)^15^. In addition, both the IGP and NCP are influenced by severe haze/fog events during wintertime^17,22^.

### Ground-based measurements of columnar aerosol properties and analysis

We analyze the measured columnar aerosol amount, composition, and aerosol radiative forcing from the AERONET^23^ (https://aeronet.gsfc.nasa.gov/) sites over Kanpur and Beijing to provide a hitherto unavailable regional and temporal picture of trends in aerosol pollution over South and East Asia. The level 2, version 3 cloud screened and quality assured daily data of aerosol optical depth (AOD) (retrieved from direct Sun algorithm), single scattering albedo (SSA), and aerosol radiative forcing (ARF) (retrieved from solar almucantar inversion algorithm) measured from the NASA Aerosol Robotic Network (AERONET) using ground-based CIMEL Sun/sky radiometers^23^ which measure direct solar and diffuse sky radiances in the spectral range of 0.34–1.02 µm, for the period of 2001–2017 over Kanpur and Beijing are utilized. The AERONET instruments are routinely calibrated approximately every 6–12 months following an elaborate procedure^23,24^ to ensure continuity and measurement quality. The data coverage is longer over Kanpur and Beijing than other sites, and they are the only two sites with longest records of the AERONET measurements with level 2 data throughout South Asia and East Asia, respectively. Over Beijing as the above data were available only for 3 months during 2001 (Mar-May), and 2005 (Oct-Dec) the data of these years are excluded in the analysis. The annual averages are calculated from the daily-average of instantaneous aerosol properties observed at 15–20 min time interval with typically about 20 measurements each day during the chosen year(s). During the monsoon season over the study region, cloud screening reduces the number of data points used for analysis. The trends are calculated using linear regression method. The linear regression method, used to derive the trends in the time series of time-dependent geographical variables, is simple, robust, and less sensitive to breaks in the time series of data. The linear regression method is appropriate when the uncertainty in data is constant (i.e. Gaussian white noise) and regression can be obtained by assigning the same precision for each parameter (or in other words allocating a weight of unity to each parameter). The statistical significance of the trends estimated for 2002–2017 is found to have a low p-value ≤ 0.05 at 95% confidence level in almost all cases.

With regard to the uncertainties in the AERONET data, the data used in this study are version 3, level 2, quality controlled, cloud screened and calibrated AERONET data, and therefore uncertainty in these parameters are not expected to change any conclusions of the study. The quality checks and associated uncertainties in the AERONET data are briefly mentioned here. The uncertainties in AODs (direct Sun) and SSA (from the inversion) depends on wavelength, for AOD it is less than ± 0.01 for wavelengths > 0.44 µm and is less than ± 0.02 for shorter wavelengths^23^ while for SSA it is ± 0.03 when the AOD at 0.44 µm is > 0.2^24^, and is expected to be more reliable for moderate to heavy aerosol loading^24^. It should be noted that the trend analysis reported here corresponds to moderate to higher aerosol loading conditions, and therefore the information from SSA at lower AODs could have been missed. The AERONET retrievals are adequately sensitive and can detect important minor features in the spectral dependence of SSA^24^. A comparison of column SSA retrieved from the AERONET data with in situ profile measurements revealed that the majority of SSA comparisons were within the uncertainty bounds when AOD at 0.44 µm was > 0.2^25^. It was also indicated that it was not possible to assess whether the AERONET retrievals were biased towards high absorption or the in situ measurements were biased low^25^. The AERONET retrieved aerosol properties have the highest accuracy for observations when solar zenith angle (SZA) is between 50° and 80°^24^, and only those data that are within this SZA range are utilized in the study. The uncertainty in calibrated sky radiance measurements of AERONET is ~ 5%^24^. The surface albedo (SA) plays a crucial role in accurately estimating aerosol radiative forcing. The effect of SA (spectral average) on aerosol radiative forcing was found to be less significant when SA was < 0.30, and was critical for aerosol radiative forcing estimates when SA was > 0.30^26^. The AERONET data is found to overestimate the ARF data at the surface (ARF_SFC_) as the upward fluxes with and without aerosols are not taken into account^26^. AERONET provides a unique value of the spectral SA for each almucantar retrieval for each location. To correct the overestimation, the AERONET ARF_SFC_ needs to be multiplied with (1-SA) where SA is the spectral average of surface albedo^26^, corresponding to Kanpur and Beijing as given in AERONET which is done in the present study, though the annual average surface albedo for Kanpur and Beijing is < 0.3. The annual mean surface albedo is 0.23 ± 0.02 for Kanpur while it is 0.15 ± 0.02 for Beijing. The seasonal mean surface albedo values for Kanpur during 2002–2003 (a whole year period chosen here as an example) are 0.22 ± 0.02 (winter), 0.23 ± 0.02 (pre-monsoon), 0.24 ± 0.02 (monsoon) and 0.22 ± 0.02 (post-monsoon), and for Beijing are 0.13 ± 0.01 (winter), 0.15 ± 0.01 (pre-monsoon), 0.15 ± 0.01 (monsoon) and 0.13 ± 0.01 (post-monsoon) respectively. The surface albedo values over the two sites do not exhibit any noticeable seasonal and inter-annual variations during 2002–2017.

Aerosol radiative forcing (ARF) depends on AOD, SSA, asymmetry parameter, surface albedo, and relative position of aerosols and clouds as a function of altitude (in case of deriving vertical profiles of ARF), and insolation. AERONET ARF values (derived for the broadband solar spectrum (0.2–4.0 µm)) are quality controlled, cloud screened and calibrated, and are retrieved for clear-sky atmosphere, and for aerosols present in the column. The AERONET measured solar fluxes were found to agree well with ground-based measurements in all situations (urban-industrial, biomass burning, mineral dust, background continental, maritime aerosols and free troposphere) with a correlation higher than 99%^27^. Further, from an analysis of year-long data, the correlation between the model estimated radiative forcing (using AOD, SSA and asymmetry parameter measured by AERONET), and AERONET radiative forcing at the top of the atmosphere (TOA) and at the Earth’s surface (SFC) was found to be very good (correlation coefficient ≥ 0.90) over an urban (Kanpur) and a rural (Gandhi College) location in the Indo-Gangetic Plain^28^, and over an urban (Pretoria) site in South Africa^29^.

ARF at the top of the atmosphere (TOA) and surface (SFC) is calculated as the change between the net (downward minus upward) flux with and without aerosols as,

1 $$\mathrm{A}R{F}_{TOA}=Flux(Net{)}_{withaerosolTOA}-Flux(Net{)}_{withoutaerosolTOA.}$$

2 $$\mathrm{A}R{F}_{SFC}=(Flux(Net{)}_{withaerosolSFC}-Flux\left(Net{)}_{withoutaerosolSFC}\right). \left(1-\mathrm{SA}\right).$$

ARF_SFC_ is multiplied by (1-SA) as mentioned above. The difference between TOA and SFC forcing is the atmospheric forcing (ATM) due to aerosols, and can be written as

3 $$\mathrm{A}R{F}_{ATM}=AR{F}_{TOA}-AR{F}_{SFC}.$$

The amount of energy absorbed by the aerosols in the atmosphere or the total radiative effect due to the presence of aerosols is given in the form of atmospheric solar heating rate (Kelvin day^−1^) as.

4 $$\frac{\partial \mathrm{T}}{\partial \mathrm{t}}=\frac{\mathrm{g}}{{c}_{p}}\left[\frac{AR{F}_{ATM}}{\Delta P}\right]*24 (\mathrm{hr}/\mathrm{day})*3600 (\mathrm{sec}/\mathrm{hr})$$

where $\partial$ T/$\partial$t is the heating rate, g is the acceleration due to gravity (9.8 ms^−2^), c${}_{\mathrm{p}}$ is the specific heat capacity of air at constant pressure (1006 J kg^−1^ K^−1^) and P is the atmospheric pressure (hPa)^3^.

The atmospheric pressure difference (ΔP) is calculated from the difference in pressure between the elevation of Kanpur (123 m)/Beijing (92 m) and 5,000 m asl. This is the common procedure for computing the aerosol heating rate using the pressure difference between the surface (altitude in m asl of study location) and 5000 m asl over the site, since most of the aerosols in the troposphere reside between the surface and 5000 m asl^5^.

### Model simulations

The trends derived from observations are compared and contrasted with the total AOD, and the species-specific extinction and scattering AOD simulated by the Modern-Era Retrospective Analysis for Research and Applications version 2 (MERRA-2) model which is based on the Goddard Earth Observing System (GEOS) atmospheric data assimilation. The MERRA-2 model uses a cubed-sphere horizontal discretization at a resolution of 0.5° × 0.625° and 72-hybrid-eta levels up to 0.01 mbar^30^. The aerosols in MERRA-2 model are simulated with a radiatively coupled version of the Goddard Chemistry, Aerosol, Radiation and Transport (GOCART) model^31^, and simulates dust, sea salt, black carbon, organic carbon (OC) and sulfate aerosols. The optical properties of the aerosol species in GOCART are based on the Optical Properties of Aerosols and Clouds (OPAC)^32^ model. The carbonaceous aerosol (BC and OC) emissions are from both natural and anthropogenic sources based on emission inventories^33^. The MERRA-2 aerosol analysis is based on the Goddard Aerosol Assimilation System^33^. The MERRA-2 aerosol assimilation system was found to show considerable skill in simulating several observed aerosol properties including surface concentrations^33,34^. SSA is calculated as the ratio of scattering AOD to extinction AOD for the total aerosol and for the respective aerosol species (sulfate (SU), black carbon (BC), organic carbon (OC), dust (DU) and sea salt (SS)) used in this study.

Sensitivity studies are conducted using the OPAC (Optical Properties of Aerosols and Clouds) model^32^ where-in AOD and SSA are simulated/derived for a specific assumed composition of aerosol types. The OPAC model is widely used for such simulations/derivations, and has been used to successfully retrieve the observed spectral aerosol optical properties (AOD, SSA and asymmetry parameter) over several distinctly different environmental settings including Kanpur^28^. As Kanpur and Beijing are densely populated urban cities with the presence of desert dust this sensitivity analysis is performed for urban aerosols with dust particles with the objective of determining the impact that any decrease and/or increase of scattering and absorbing aerosols would cause on aerosol properties (content and composition). This can be used to support mitigation efforts by analyzing what kinds of changes in columnar aerosol loading (in terms of AOD) would result from various reductions in the various aerosol components, and how this differs regionally. The urban aerosols are comprised of 56.0 µg m^−3^ of water soluble species (sulfate, nitrate and organic aerosol species), 35.6 µg m^−3^ of insoluble particles (soil particles with a certain amount of organics) and 7.8 µg m^−3^ of BC^32^. Along with the urban aerosols, 27.8 µg m^−3^ of dust particles (1000 particles per cm^3^) are added to represent the aerosols observed over Kanpur and Beijing. The aerosols are distributed exponentially with altitude on the basis of aerosol scale height, which depicts the slope of the aerosol profile, ca. 8 km for the urban aerosols^32^. The layer thickness for all aerosol types (water soluble, insoluble and BC) in urban aerosols and the dust particles (the minimum and maximum boundaries of the aerosol layer) is prescribed to be 2 km^32^.

## Results and discussion

### Satellite observations of Asian aerosol dipole

**Figure 2:**
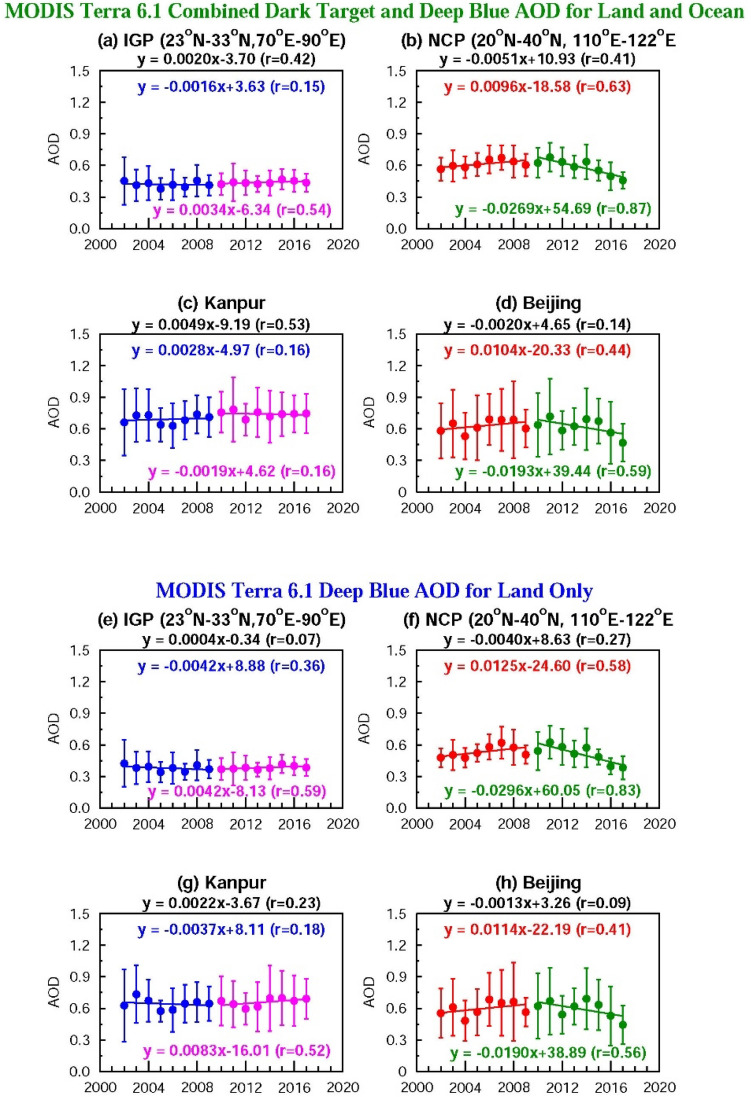
Trends in satellite retrieved AODs over South and East Asia: Area-averaged AOD (MODIS Terra v 6.1 Combined Dark target and Deep Blue) trends for (**a**) IGP in South Asia (p-value 0.69 (2002–2009), 0.19 (2010–2017) and 0.14 (2002–2017) at 95% confidence level (CL)) and (**b**) NCP in East Asia (p-value 0.10 (2002–2009), < 0.01 (2010–2017) and 0.09 (2002–2017) at 95% CL) (for regions shown as boxes in Fig. 1). The trends for AOD data over the grids where Kanpur (p-value 0.74 (2002–2009), 0.64 (2010–17) and 0.05 (2002–17)), and Beijing (p-value 0.29 (2002–2009), 0.12 (2010–2017) and 0.55 (2002–2017)) are located are shown in (**c**) and (**d**). Area-averaged AOD (MODIS Terra v 6.1 Deep Blue) trends for (**e**) IGP in South Asia (p-value 0.36 (2002–2009), 0.13 (2010–2017) and 0.89 (2002–2017) at 95% confidence level (CL)) and (**f**) NCP in East Asia (p-value 0.13 (2002–2009), < 0.01 (2010–2017) and 0.28 (2002–2017) at 95% CL) (for regions shown as boxes in Fig. 1). The trends for AOD data over the grids where Kanpur (p-value 0.67 (2002–2009), 0.19 (2010–2017) and 0.46 (2002–2017)), and Beijing (p-value 0.32 (2002–2009), 0.14 (2010–2017) and 0.69 (2002–2017)) are located are shown in (**g**) and (**h**). Vertical bars correspond to ± 1σ (standard deviation) from the mean.

Satellite observations clearly show a regional-scale dipole, as well as the decadal-scale changes in aerosol content (AOD) as well as composition (SSA) (Fig. 1). Trends in AOD based on satellite data for the region (Fig. 1) as well as over the two locations of Kanpur and Beijing (Fig. 2) for the 2002–2017 period are robust and similar—AOD increases over South Asia (boxed area) and Kanpur (almost no trend), and decreases over East Asia (boxed area) and Beijing (significant) (Fig. 1). The combined AODs are marginally higher than the Dark Target AODs. The spatial patterns in level-2 and level-3 SSA and their differences are similar; SSA values are slightly lower in level-2 data, consistent with sensitivity and retrieval errors^20^. The SSA was slightly overestimated when they were > 0.92 and were underestimated when they were more absorbing, and the error increased with decreasing SSA^20^. The ± 1σ (standard deviation) from the mean (vertical bars) in area-averaged AODs decreases in the more recent years (Fig. 2a,b,e,f) indicating a regional homogeneity in increase or decrease of aerosol emissions. The trends are similar in both MODIS AOD products on local and regional scales (Fig. 2) (except over Kanpur where AODs from the Deep Blue algorithm, and the Combined Dark Target and Deep Blue algorithm exhibit differing trends for the 2002–2009, and the 2010–2017 periods). A contrasting feature in trends is notable between the two regions—the rate of increase is higher at local scale (Kanpur) in South Asia when compared to the region (Fig. 2a,c,e,g), while over East Asia the rate of decrease is greater over the region compared to the local scale of Beijing (Fig. 2b,d,f,h). However, although there are differences in the magnitudes of rates of increase/decrease on regional and local scales, based on the overall similarity in aerosol characteristics (as discussed in “Methods”) and in the trends we make use of the two individual locations as being broadly representative of the respective regions.

### Aerosol optical depth (AOD) over Kanpur and Beijing

**Figure 3:**
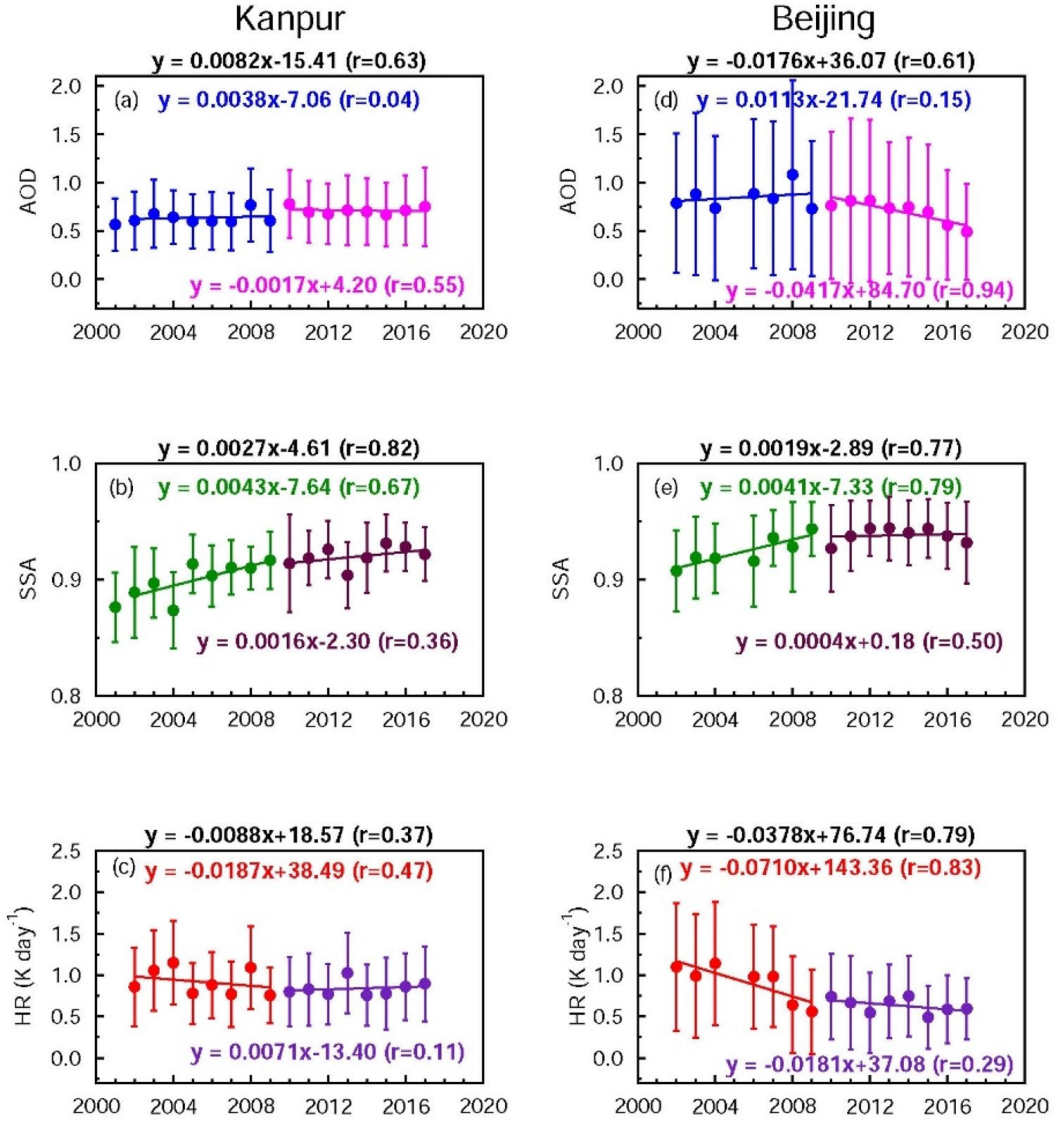
Aerosol optical properties and heating rate over Kanpur and Beijing during 2001–2017 derived from the AERONET observations: Annual mean (**a**, **d**) aerosol optical depth (AOD) and (**b**, **e**) single scattering albedo (SSA) over Kanpur and Beijing, respectively, at a wavelength of 0.50 µm. (**c**, **f**) atmospheric solar heating rate due to aerosols (HR) (K day^−1^) estimated using the aerosol radiative forcing (Wm^−2^) of the atmosphere over Kanpur and Beijing. Vertical bars indicate ± 1σ (standard deviation) from the mean. Trend lines and equations are drawn for two different periods: 2002–2009, and 2010–2017 respectively. The equations for the overall trend (2002–2017) for the respective aerosol parameter are given (in black) at the top of each figure.

Over almost two decades since 2001, AOD increased over Kanpur and decreased over Beijing (Fig. 3), thus clearly revealing diverging trends in the columnar aerosol loading (Fig. 3). The annual average AOD levels over Beijing were higher (0.73–1.08) than over Kanpur (0.57–0.77) during the first decade of the twenty-first century (2002–2009). In the next decade (2010–2017) this dipole in the AODs was maintained but reversed: over Kanpur the AOD (0.68–0.78) has become higher than over Beijing (0.49–0.81). AODs increased over both South and East Asia during the previous decade (2002–2009), the rate of increase being ca. 3 times higher over East Asia than South Asia. The 2008 Olympics Games held in China was a pivotal event after which more stringent pollutant controls were put in place. Subsequently, because of the reduction in emissions of primary aerosols and aerosol precursor gases as a result of comprehensive emission controls in China, the AOD has been sharply decreasing during the current decade (2010–2017), 25-times faster over East Asia than South Asia (Fig. 3). The other major notable difference arises in the intra-annual variability of AODs—the intra-annual variation in AOD (revealed by the vertical bars denoting ± 1σ standard deviation from the mean in Fig. 3) is higher over Beijing than Kanpur, and it remains within a narrow range over Kanpur (± 0.30 to 0.40) during the last two decades while over Beijing it is higher during 2002–2009 (± 0.69 to 0.98) than the last decade (± 0.49 to 0.85) (Fig. 3). The variation in the standard deviation over these regions is due to differences in the amounts and changes in regional aerosol emissions^11,13^, as the effects of inter-annual meteorological variations on PM_2.5_ concentrations during 2013–2017 were relatively small^35^.

### Single scattering albedo (SSA) over Kanpur and Beijing

The single scattering albedo (SSA), a measure of absorption capacity of aerosols, which is related to aerosol composition, has increased over Kanpur and Beijing in the last two decades, suggesting that aerosol composition has changed over Asia, i.e., the aerosols have become more scattering in nature (Fig. 3). The rates of increase in SSA are similar over South Asia and East Asia during the previous decade, while in the current decade the rate of increase has decreased, with the rate of increase being higher over South Asia (Fig. 3). The SSA starts in 2002 being significantly higher over Beijing (0.91) than Kanpur (0.88), and becomes nearly the same towards the end of two decades (0.93), an increase of 5.7% over Kanpur and 2.2% over Beijing. The aerosol emissions over South and East Asia have been changing since 2008 due to the introduction of stringent air quality measures, more so in East Asia, aimed to address the public health concerns. The reduction of sulfur dioxide (SO_2_)^13^, and other pollutants including black carbon (BC) is seen to be more rapid over China^11^. The increase in SSA (ratio of scattering to extinction), can be brought about by an increase in scattering aerosols (e.g., sulfate), and/or a decrease in absorbing aerosols (e.g., BC), as the ratio of BC-to-sulfate influences the SSA. It should be noted that in this study the trends in SSA, which depicts the net effect of influence of the increase/decrease in scattering/absorbing aerosols to the total columnar chemical composition, are derived. Over China emissions of both SO_2_ and BC have decreased^13^. Significant differences in their rates of decrease—SO_2_ emissions decreased by 62% and BC by 28% during 2010–2017^11^ resulted in increased SSA values over East Asia, and the increase in SSA over South Asia can be mainly attributed to increasing SO_2_ emissions^13^. It may be noted that not only BC emissions decreased by 25%, but the BC-to-sulfate ratio, an important determinant of SSA, also decreased by 20% over Beijing, and thus sulfate altered aerosol absorption properties^36,37^ which could have resulted in an increase in the SSA. It was found that in a sulfate-dominated environment, SSA enhanced with gradual increase of sulfate with increase in ambient relative humidity, and absorption of BC and brown carbon (BrC) got altered through the internal mixing of aerosols^37^. In addition, the relative contribution of fossil fuel and biomass fuel burning to BC emissions differs between East and South Asia; the fossil fuel contribution to the total BC is 60–80% over East Asia, whereas it is only about 30–50% over South Asia^36,38,39^.

### Seasonal trends in AOD and SSA

**Figure 4:**
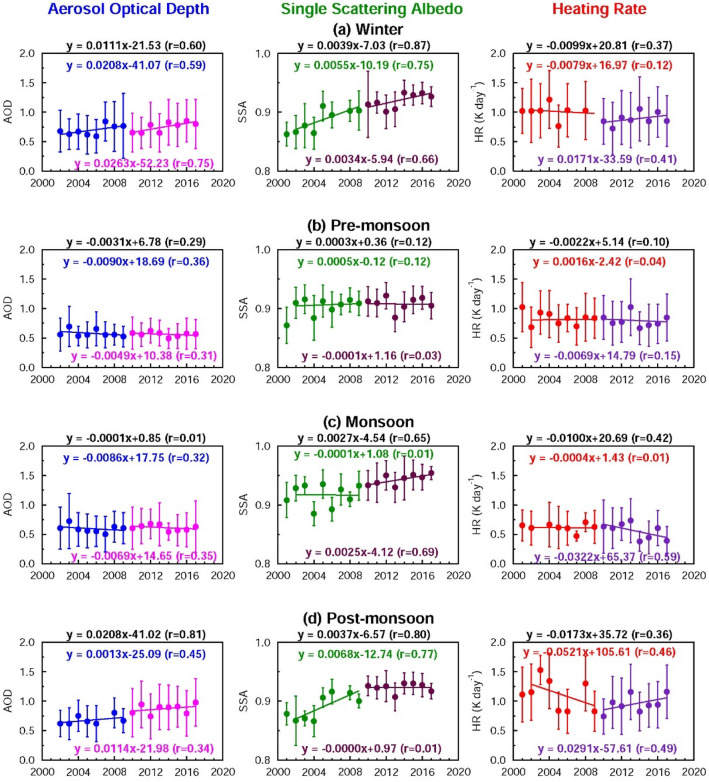
Seasonal aerosol optical properties and heating rate over Kanpur during 2002–2017 derived from the AERONET observations: Seasonal trends in aerosol optical depth (AOD) and single scattering albedo (SSA) and atmospheric solar heating rate due to aerosols (HR) (K day^−1^) for (**a**) winter, (**b**) pre-monsoon, (**c**) monsoon and (**d**) post-monsoon respectively. Vertical bars indicate ± 1σ (standard deviation) from the mean. Trend lines and equations are shown for two different periods: 2002–2009, and 2010–2017 respectively. The equations for the overall trend (2002–2017) for AOD, SSA and HR are given (in black) at the top of each figure.

**Figure 5:**
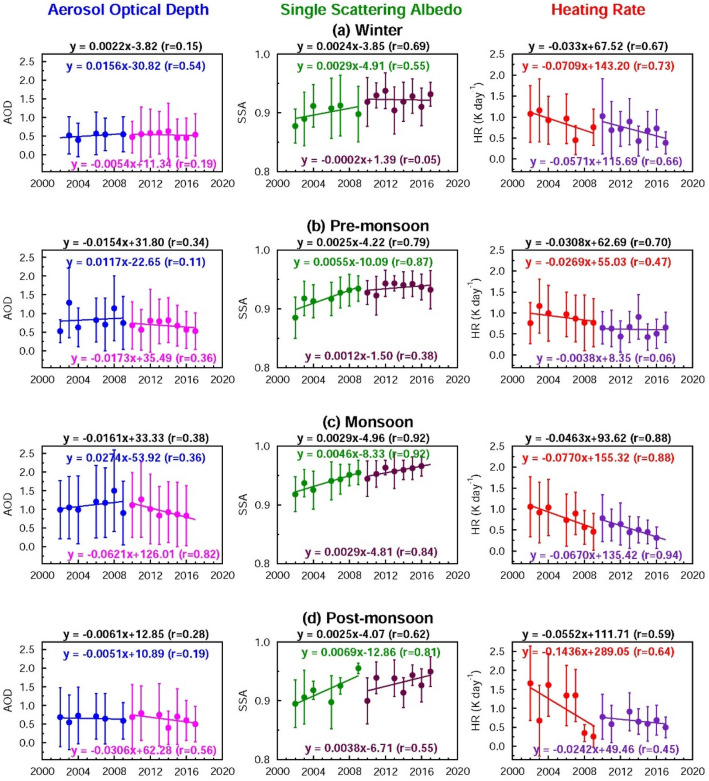
Seasonal aerosol optical properties and heating rate over Beijing during 2002–2017 derived from the AERONET observations: Seasonal trends in aerosol optical depth (AOD) and single scattering albedo (SSA) and atmospheric solar heating rate due to aerosols (HR) (K day^−1^) for (**a**) winter, (**b**) pre-monsoon, (**c**) monsoon and (**d**) post-monsoon respectively. Vertical bars indicate ± 1σ (standard deviation) from the mean. Trend lines and equations are shown for two different periods: 2002–2009, and 2010–2017 respectively. The equations for the overall trend (2002–2017) for AOD, SSA and HR are given (in black) at the top of each figure.

So far our focus has been on deriving annual trends. It is also imperative to examine the seasonal trends over both sites in order to attribute annual trends to seasonal aerosol loading. Both regions undergo seasonal changes in aerosol amounts and types i.e., due to seasonal changes in anthropogenic activities (“Satellite observations over Asia” section). Thus, decomposition of annual trend into seasonal trends in both AOD and SSA would allow different sources of pollution responsible for seasonal and annual trends in aerosols to be delineated. The annual increasing trends in AOD and SSA are controlled by trends during winter and post-monsoon seasons over Kanpur (Fig. 4, Table 1) which are dominated by anthropogenic aerosol emissions. SSA exhibits a statistically significant increasing trend in winter during 2002–2009, and during the monsoon periods of 2010–2017. The increase in AOD during post-monsoon and winter suggest an increase in the anthropogenic aerosol loading over the IGP. An analysis of the multi-year time series of MODIS Aqua Multi-Angle Implementation of Atmospheric Correction (MAIAC) AODs at 0.47 µm over the IGP region showed an increase in AOD of 0.0187 per year during the post-monsoon and 0.0168/year during the winter over the IGP^40^ during 2002–2016; this increase was attributed to an increasing trend in agricultural fires during the same period as a major factor contributing to the trend, in addition to the increase in fossil fuel burning emissions. The AODs over Kanpur in the IGP increased by 0.0208/year and 0.0111/year during 2002–2017 in the post-monsoon and winter, seasons respectively (Fig. 4). The results from the present study compare well with results from ref. 40. The increasing trend in AOD is higher during the post-monsoon than during winter in both studies. The MODIS-MAIAC AODs for 2002–2016 over the IGP were found to show increasing trends during the pre-monsoon and monsoon seasons; however, the trends were significantly lower at 0.0046/year and 0.0070/year, respectively^40^. In contrast, the AODs during the pre-monsoon and monsoon exhibit decreasing trends over Kanpur in the current study (Fig. 4b,c). The differences in trends between the present study and ref^40^ could arise due to differences in spatial coverage (point (Kanpur) vs. wide area (IGP)), time period (2002–2017 for Kanpur where as it was 2002–2016 for the IGP in ref. 40), technique (in situ ground based in case of Kanpur while the AODs are derived from satellite for IGP) and retrieval algorithms. Over Beijing a statistically significant decreasing trend in the monsoon periods during 2010–2017 controls the annual trend (Figs. 3, 5, Table 1). The changes in SSA during the entire two decades are statistically significant on seasonal and annual scales over Beijing, suggesting a robust overall increase in the scattering nature of aerosols. The SSA trend during the monsoon is statistically significant during 2002–2009, 2009–2017 and 2002–2017 (Table 1). The vegetation fires in South and Southeast Asian countries have shown an increasing trend^41^, however, there are no studies reporting trends or changes in biomass burning activities such as agro-residue/waste burning and forest fires over East Asia which might influence aerosol loading and composition. In the absence of such information it is clear that over Beijing the reduction in aerosol content (mainly anthropogenic sulfate, and BC), and decrease in BC emissions lead to a decrease in AOD and increase in SSA in the last decade. Thus, the seasonal trend analysis clearly illustrates that natural aerosols (dust and sea salt) did not change significantly over the IGP (AODs show a slight decreasing trend in the northwestern part of the IGP during the pre-monsoon and monsoon seasons^40^ which are influenced by the presence of dust and sea salt; sea salt particles contribute ≤ 10% to the AOD during the monsoon^17^ when their atmospheric abundance is highest), and the NCP during the last two decades enough to alter the AOD and SSA, whereas the changes (increase/decline) in AOD and SSA are brought about mainly by the increase/decrease in emissions of anthropogenic aerosols and aerosol precursor gases.

**Table 1:** Details of statistical tests (p-values at 95% confidence level) for the trends in aerosol optical depth (AOD), single scattering albedo (SSA) and aerosol-induced atmospheric heating rate (HR) over Kanpur and Beijing on seasonal and annual scales. p-values ≤ 0.05 are highlighted in boldface.

**Season** Period	Kanpur			Beijing		
	AOD	SSA	HR	AOD	SSA	HR
**Winter**						
2002–2009 2010–2017 2002–2017	0.13 **0.03** **0.02**	**0.05** 0.07 ** < 0.01**	0.81 0.33 0.17	0.36 0.63 0.67	0.33 0.73 **0.02**	0.09 0.07 ** < 0.01**
**Pre-monsoon**						
2002–2009 2010–2017 2002–2017	0.36 0.43 0.23	0.97 0.78 0.77	0.94 0.70 0.65	0.82 0.37 0.20	**0.02** 0.55 ** < 0.01**	0.28 0.88 ** < 0.01**
**Monsoon**						
2002–2009 2010–2017 2002–2017	0.42 0.37 0.89	0.88 0.10 **0.02**	0.96 0.12 0.11	0.43 **0.02** 0.17	** < 0.01** **0.03** ** < 0.01**	** < 0.01** ** < 0.01** ** < 0.01**
**Post-monsoon**						
2002–2009 2010–2017 2002–2017	0.32 0.43 ** < 0.01**	**0.05** 0.72 ** < 0.01**	0.29 0.23 0.17	0.70 0.19 0.33	0.06 0.26 **0.05**	0.12 0.30 **0.02**
**Annual**						
2002–2009 2010–2017 2002–2017	0.99 0.25 **0.01**	0.19 0.59 ** < 0.01**	0.15 0.90 0.06	0.59 ** < 0.01** 0.15	0.27 0.23 **0.04**	0.11 0.23 ** < 0.01**

### Model simulations

**Figure 6:**
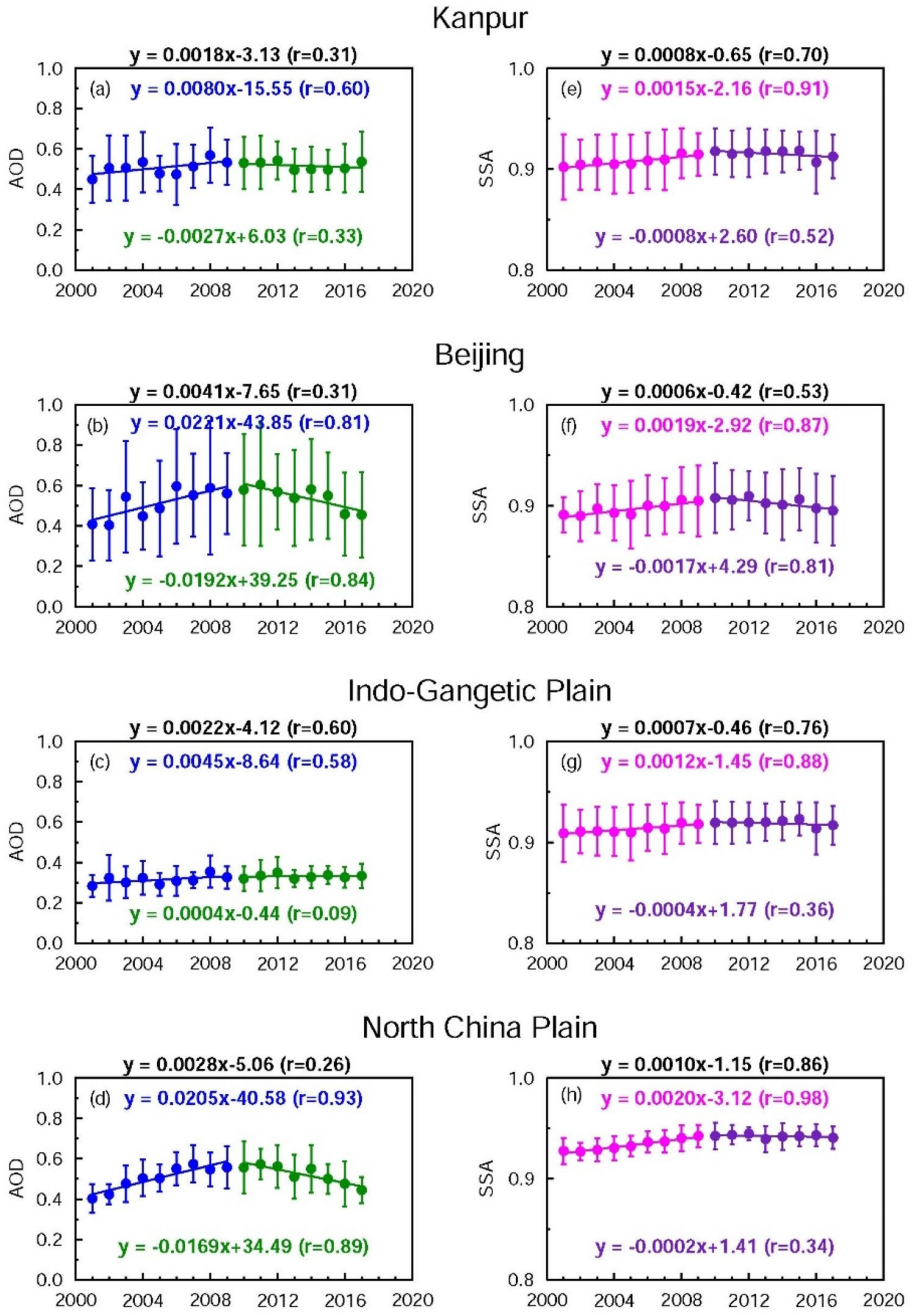
Model (MERRA-2) simulated aerosol optical properties over the IGP and NCP during 2002–2017: Trends in annual mean aerosol optical depth (AOD) and single scattering albedo (SSA) over Kanpur (**a**, **e**) and Beijing (**b**, **f**), respectively, corresponding to a wavelength of 0.55 µm. Trends in AOD and SSA are shown for the IGP (**c**, **g**) and the NCP (**d**, **h**), respectively. Vertical bars correspond to ± 1σ (standard deviation) from the mean. Trend lines and equations are drawn for two different periods: 2002–2009, and 2010–2017 respectively. The equations for the overall trend (2002–2017) for the respective aerosol parameter are given (in black) at the top of each figure.

**Figure 7:**
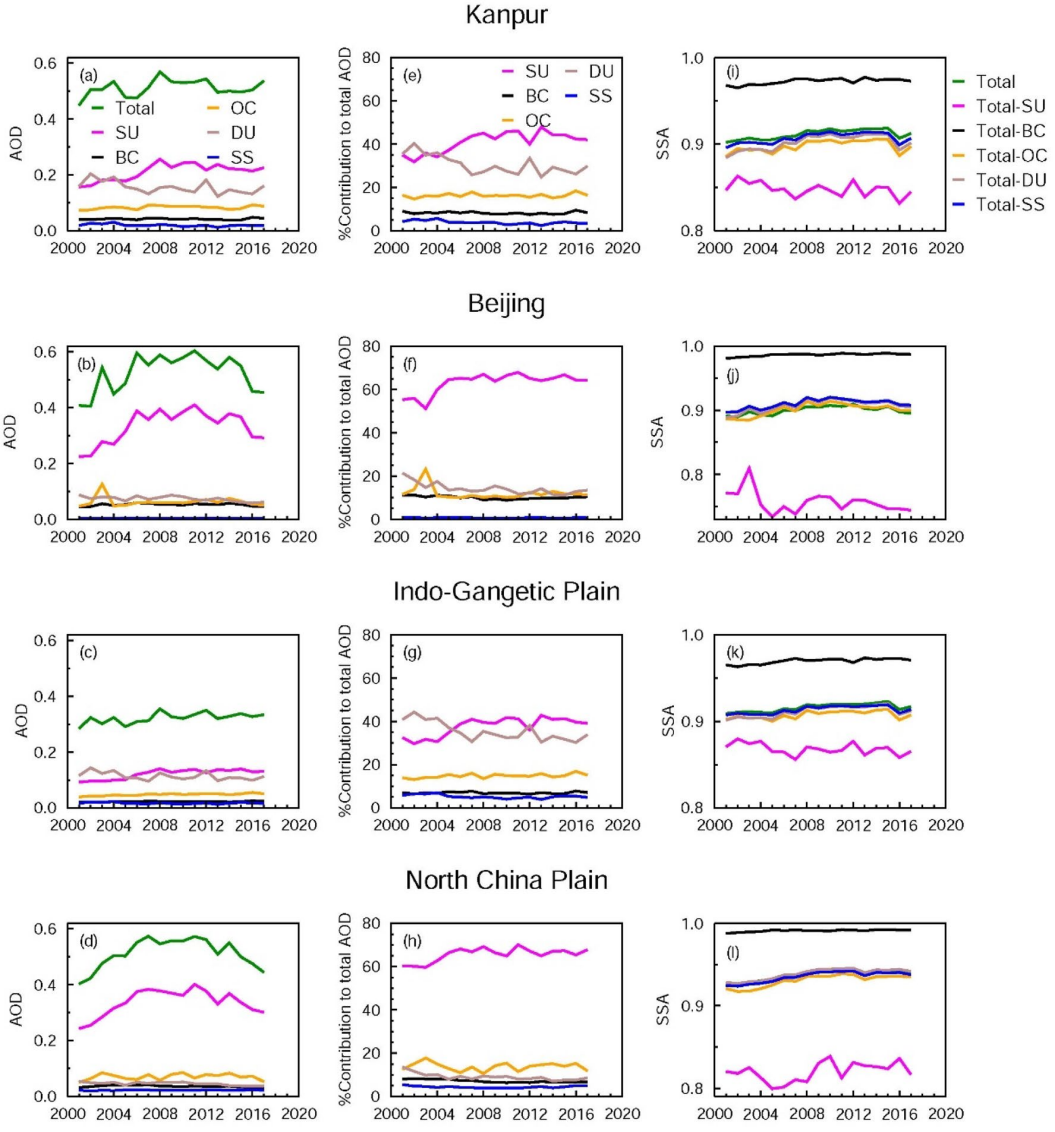
Model (MERRA-2) simulated optical properties of aerosol species over the IGP and NCP during 2001–2017: Total AOD, and species AODs—sulfate (SU), black carbon (BC), organic carbon (OC), dust (DU) and sea salt (SS) over (**a**) Kanpur, (**b**) Beijing, (**c**) Indo-Gangetic Plain (IGP) and (**d**) North China Plain (NCP). Percentage contribution of SU, BC, OC, DU and SS to the total AOD during 2001–2017 over (**e**) Kanpur, (**f**) Beijing, (**g**) IGP and (**h**) NCP. Single scattering albedo (SSA) of total (composite) aerosol, and SSA obtained by subtracting the SSA of sulfate, black carbon, organic carbon, dust and sea salt respectively from the total SSA are plotted for (**i**) Kanpur, (**j**) Beijing, (**k**) IGP and (**l**) NCP.

At the outset, the MERRA-2 model is able to simulate the trends in AODs well over South and East Asia (Kanpur, Beijing, IGP and NCP); however, the model simulated AODs (Fig. 6) are lower than MODIS (Fig. 2) and AERONET (Fig. 3) AODs. The decreasing trend in AOD over Beijing and NCP during 2010–2017 is very well captured by the model, as well as the slight declining tendency in AOD over Kanpur and the IGP. Overall, the AODs over Kanpur and the IGP increase during 2002–2017 with trends of a similar magnitude—0.0018/year and 0.0022/year for Kanpur and the IGP, respectively (Fig. 6), which compares well with an increasing trend reported for different seasons derived using MODIS Aqua MAIAC AODs over the IGP for 2002–2016^40^. The model is able to capture the overall increasing trend in SSA over Kanpur and Beijing for the 2002–2017 period; however, the rates are lower than observations. Further, the model is also able to reproduce the observed increases in SSA during the first decade (2002–2009) over Kanpur and Beijing, however, the model is unable to capture the increasing trend in SSA seen in the observations for the time period 2010–2017. The model SSA shows a slightly increasing trend on regional scales as well (IGP, NCP) during 2002–2017. An analysis of contributions of different aerosol species to total AOD and SSA presents interesting features over the IGP and NCP. Over Kanpur and IGP the AOD is dominated by almost equal contributions from sulfate and dust (~ 35–40%), whereas sulfate (~ 60%) dominates the total AOD over Beijing and the NCP (Fig. 7), followed by organic carbon. The contributions by BC and sea salt particles to AOD are less (< 15%) over both regions. The MERRA-2 simulated AODs are lower, and SSA values are higher than those derived from the AERONET observations. This underestimation of contribution by BC particles to the total AOD results in higher SSA values. These MERRA-2 simulated features in aerosol species are quite similar to those reported previously from GOCART and MOZART model simulations over Kanpur, the IGP and India^17^. The model-observation differences in aerosol properties were attributed to the lack of/absence of proper aerosol emissions and removal mechanisms in the models^14,17^, and called for improving the emission inventories of dust sources, region specific fossil fuel and biomass emission sources (biomass burning, forest fires and open burning of crop waste). Sulfate and BC AOD start to decrease from 2009 over Beijing and the NCP. SSA increases (more scattering) when BC is removed and it decreases significantly (more absorbing) when sulfate aerosols are removed from the atmosphere (Fig. 7); however, SSA does not change significantly when organic carbon, dust and/or sea salt aerosols are removed from the atmosphere because their SSA values as derived from MERRA-2 are nearly 1 (0.98, 0.93 and 1.00 respectively at 0.55 µm). The SSA of pure sulfate and BC are 1.00 and 0.23 respectively. Though the SSA of sulfate is equal to 1, the SSA of total aerosol changes significantly because the contributions (in terms of amount) of a mixture of various species determine the SSA of the mixture even if sulfate represents the largest contribution to the total aerosol loading, i.e., AOD (Fig. 7).

These findings are further strengthened by a sensitivity study using the OPAC model. Reducing the water soluble species by 60% while keeping BC unchanged results in 32% and 11% reductions in AOD and SSA (Table 2), respectively. In addition, when reducing BC by 25%, the reduction in AOD and the change in SSA is negligible (Table 2). These simulations using OPAC are conducted mimicking the observed decreases^11,13^ in emissions of the respective aerosol species to determine the impact these reductions in emissions have on AOD and SSA.

**Table 2:** Aerosol optical depth (AOD) and single scattering albedo (SSA) for aerosols over urban regions corresponding to different scenarios. AOD and SSA correspond to 0.50 µm wavelength, and for 50% relative humidity. Change (in percentage) in AOD and SSA with respect to Case 1—Urban aerosols with dust are given (changes are rounded off to whole numbers).

Case	AOD	SSA	% change w.r.t. case 1 in	
			AOD	SSA
1. Urban aerosols with dust (case 1)	0.69	0.80	–	–
2. Case 1—60%WS	0.47	0.71	− 32	− 11
3. Case 1—25%BC	0.65	0.83	− 6	4
4. Case 1—(60%WS, 25%BC)	0.43	0.76	− 38	− 5
5. Case 1—50%BC	0.61	0.87	− 12	9
6. Case 1—(60%WS, 50%BC)	0.40	0.81	− 42	1
7. Case 1—dust	0.55	0.76	− 20	− 5

Further reducing BC by 50% (instead of 25%), the changes in AOD and SSA are linear and are twice those in the scenario with 25% reduction in BC (Table 2). As a function of relative humidity (RH) for RH < 50% (> 50%) the decrease is greater (less) for AOD and the increase is less (greater) for SSA than that for an RH of 50%. The reduction in dust also results in a notable decrease in AOD (20%); however, the change in SSA is small (− 5%), as seen earlier in results from model simulations, suggesting that reducing dust particles leads to a decrease in the loading, while overall the aerosols become more absorbing. Thus, results from MERRA-2 model (Fig. 7), and the OPAC model simulations (Table 2) clearly show that in order to reduce the columnar aerosol loading (in terms of AOD), sulfate and its precursors have to be substantially reduced, whereas in order to make the aerosols more scattering in nature (i.e., an increase in SSA), BC needs to be reduced more significantly. Among the scenarios presented here in Table 2, the results of Case 6 mimic closely the observations (significant reduction in AOD accompanied with a moderate increase in SSA). The situation over East Asia (a 38% reduction in AOD, and a small increase of 0.02 in SSA) and South Asia (a 24% increase in AOD and a moderate increase of 0.04 in the SSA) (Table 2) calls for more significant reductions of BC over East Asia, whereas both sulfate and BC need to be reduced over South Asia, which in turn will reduce atmospheric heating on a regional scale over both regions. Furthermore, secondary organic aerosols, which comprise up to 70% of the aerosol concentration in metropolitan areas, have been increasing over China^43^. The decreases in SO_2_ and BC emissions can also change the proportion of secondary organic aerosols, which are scattering in nature, thereby increasing the SSA.

### Atmospheric solar heating rates due to aerosols

The aerosol-induced atmospheric heating (HR) has decreased over both South and East Asia during the last two decades (Fig. 3). The decrease of HR is mostly due to the increase in SSA, which outweighs the small increase in AOD during 2002–2009 (Fig. 3). The aerosol radiative forcing of the atmosphere, which is used to derive the HR, is dependent on the value of SSA—a lower SSA is associated with a higher atmospheric warming and vice versa. The HR is higher over South Asia than East Asia because of the lower SSA. The relationship between AOD and atmospheric warming is linear, while between SSA and atmospheric warming it is non-linear; for example, HR increases by 10% for a 10% increase in AOD, but it increases by > 20% for a 10% decrease in SSA from 0.99 to 0.89. The HR decreases to < 0.6 K day^−1^ over Beijing towards the end of the current decade (Fig. 3), while it has remained consistently > 0.8 K day^−1^ every year during the two decades over Kanpur. The annual mean HR is consistently high and regionally coherent for almost two decades over Asia. Though the HR is currently lower than in the last decade, it is still ≥ 0.6 K day^−1^ (East Asia) and > 0.8 K day^−1^ (South Asia) (Fig. 3), which has significant climate implications. The HR (Fig. 3) is comparable to the HR obtained in spring 2007 for the plumes from Beijing (East Asia)^36^, and at least 1.5-times higher than the reported value in the range of 0.45–0.7 K day^−1^ during spring 2006 in the outflow from India over the Maldives in the Indian Ocean^3^. We have compared the results that are relevant and available over the region covering South and East Asia, including the Indian Ocean, IGP and the Himalayas. The earlier studies^3,36^ were based on limited sets of measurements made during a particular season and/or over a particular location. Surface and aircraft measurements of BC and radiation made during Aug-Sep 2008 over Beijing, Shanghai and Yellow Sea were used to derive vertical profiles of HR^36^. Vertical profile measurements of aerosol absorption and scattering coefficients in the 0–3 km altitude range, and a Monte Carlo Aerosol-Cloud Radiation (MACR) model were used to derive aerosol heating rates over the Maldives, though only during the pre-monsoon season^3^. Our study reports—for the first time—the trends in AOD, SSA and HR derived from AERONET over Kanpur (South Asia) and Beijing (East Asia) using the data from 2001 to 2017.

Overall HR decreases over both locations (Fig. 3c,f); however, the rate of decrease over Beijing is more rapid (> 3-times faster) than over Kanpur. The HR decreases over East Asia 3-times faster than over South Asia in the previous decade (2002–2009), while during the current decade (2010–2017) the HR decrease slows down over Beijing, whereas there is no notable change over Kanpur (Fig. 3). The HR decrease is lower in the current decade despite a significant decrease in AOD (especially over East Asia). This occurs mainly because SSA has been increasing and the effect on HR is strongly non-linear at high SSA values. If the increasing trend in SSA in this decade had continued at the same rate as in the previous decade (Fig. 3), then the aerosols would have become much more reflective, and the decrease in HR would have been significantly higher. This is due to the fact that though the column content of aerosols (AOD) decreased significantly, SSA (composition) (Figs. 1, 3) did not change as much as the AOD, as there were significantly different relative rates of decrease of scattering (e.g., sulfate) and absorbing (e.g., BC) aerosols^11^, as seen earlier. The annual HR trends over South Asia are dominated by the increasing trends during winter and post-monsoon due to an increasing (decreasing) AOD (SSA), while the HR decreased during monsoon primarily due to an increase in SSA during 2010–2017 (Fig. 4) as the AOD values during the monsoon are more or less similar. Over East Asia the annual decreasing trend in HR during 2002–2017 is contributed by the significant decreasing trends in HR in all the seasons (Table 1, Fig. 5); the decreasing trends in HR are significant for all the time periods during the monsoon (Table 1).

The changes in aerosol emission patterns resulting in changes in AOD, SSA and HR exhibit variations on inter- and intra-decadal time-scales (Table 3, Fig. 3). Overall AOD increased by 24% over Kanpur, SSA and HR increased by 4% in 2017 when compared to 2002 (Table 3). During the same period AOD decreased by 38%, SSA increased by ~ 3% and HR decreased by 46% over Beijing. The variations in AOD and HR are significantly higher than SSA (Table 3) between 2010–2017 and 2002–2009. AOD increased by 12% over Kanpur, whereas it decreased by 17% over Beijing, while the SSA increased over both locations, although the change is small (1.5–2.1%) (Table 3). The HR decreased significantly over Beijing (4-times more) than over Kanpur. The satellite observations of both regions (shown in Fig. 1) show that AOD increased by ~ 5% over the IGP (black box) in South Asia and decreased by the same amount over the North China Plain (black box) in East Asia between the above two periods, confirming that the changes in aerosol loading are of regional scale. On the intra-decadal scale (difference between 2014–2017 and 2010–2013) the decrease in AOD is significantly higher over Beijing (− 20%). These variations from in-situ ground-based AOD measurements over two aerosol hotspot regions in South and East Asia are consistent with the satellite observed changes in AOD over these regions (Fig. 1, AOD increased by 5% over IGP and decreased by 15% over NCP), revealing a divergence in the trends of aerosol loading (AOD) over these two regions. SSA increased by about the same absolute amount over both regions; however, the relative rate of change is different, being lower in East Asia, which can be attributed to the different rates of decreases of SO_2_ and BC emissions^11^. The HR decreased by 8% during 2014–2017 when compared to 2010–2013 over East Asia, a factor of 2 more than over South Asia. These trends of aerosol content and composition, obtained for the first time over South and East Asia, clearly show the significant radiative impacts that changing aerosol emissions have on the atmosphere on decadal and intra-decadal time scales.

**Table 3:** Changes in aerosol optical depth (AOD), single scattering albedo (SSA), and heating rate (HR, K day^−1^) for three time periods (total, decadal, and intra-decadal) over Kanpur and Beijing (rounded off to 2 decimal digits). Numbers in parenthesis represent the change in % (rounded off to 1 decimal digit).

Change period	Kanpur			Beijing		
	ΔAOD	ΔSSA	ΔHR	ΔAOD	ΔSSA	ΔHR
2017–2002	0.14 (23.8)	0.03 (3.7)	0.04 (4.2)	− 0.30 (− 37.7)	0.02 (2.7)	− 0.50 (− 45.9)
(2010–2017)–(2002–2009)	0.07 (11.6)	0.02 (2.1)	− 0.08 (− 8.5)	− 0.15 (− 17.2)	0.01 (1.5)	− 0.28 (− 30.8)
(2014–2017)–(2010–2013)	− 0.01 (− 1.1)	0.01 (1.0)	− 0.03 (− 3.8)	− 0.16 (− 20.2)	0.01 (0.1)	− 0.06 (− 8.3)

## Conclusions

The climate responses to the changing aerosol patterns (loading, composition and radiative effect) over Asia are highly uncertain^1^. Over the Asian region the model simulations of the monsoon due to aerosol perturbation, and the representation of precipitation are uncertain^1^. The diverging aerosol trends (Figs. 1–5) seen over Asia introduces yet another layer of complexity in aerosol-climate interactions over Asia. It is clear from the present analysis that an accelerated reduction in aerosol emissions, notably light-absorbing aerosols, contributes to reducing the atmospheric solar heating rates due to aerosols in these regions, in addition to major health benefits. It is equally important to realize that the reduction in aerosol emissions leads to a reduction in the column content of aerosols (AOD); however, the composition (SSA) is also simultaneously changing (Figs. 1, 3). Thus, it is important to know which type(s) (amount and proportion) of aerosol species to prioritize for mitigation, i.e., what will result in the most significant decrease in column content and atmospheric warming. This underlines the emergent need to reduce SO_2_ emissions accompanied with larger reduction in BC emissions, resulting in an increase in the contribution of scattering aerosols (higher SSA), which will result in both surface cooling and a reduction in atmospheric heating. Primary as well as secondary aerosols both need to be reduced to account for the complex interactions between the different pollutant types. However, in the context of changing aerosol regimes, though the primary aerosols are observed to be decreasing, the proportions of secondary aerosols are increasing^43^. There are also complex effects of changes in aerosols on gas phase chemistry. For example, surface ozone, which is harmful to human health and plants, and is produced in polluted air by photochemical oxidation of volatile organic compounds (VOCs) in the presence of nitrogen oxides (NO_x_), is increasing over China^44^. This increase in surface ozone was deduced to be due to the decrease in fine particulate matter, which slows down the aerosol particle sink of hydroperoxy radicals, and in turn leads to enhanced ozone production^44^. This needs to be taken into account in the case of South Asia as well, where both particulate matter and ozone are expected to increase in the coming decades^45^.

Given that the situation in terms of aerosol loading over East Asia was relatively worse than South Asia around the year 2000, the progress made in East Asia towards reducing aerosol loading and resultant atmospheric heating up to the present is encouraging. This provides hope for South Asia in calling for similarly aggressive efforts towards reducing air pollution and atmospheric warming. Further analyses such as this, along with model simulations, will be valuable in unravelling the aerosol-climate interactions over Asia, and helping to reduce their impacts on both climate and human health. There is a clear need for further studies, in particular, extensive simulations with climate models to understand any implications of the diverging trends in aerosols between East Asia and South Asia and how this might contribute to climate change, i.e., slowing down or unmasking the global warming in the near term, as well as possibly contributing to large-scale changes in regional atmospheric stability, circulations and hydrological cycles in both regions. Follow-up studies to examine these issues would be of value for better understanding the expected future climate impacts and the potential value of various mitigation measures.

## Data Availability

All data used in the manuscript are publicly available at https://giovanni.gsfc.nasa.gov/giovanni/, https://disc.gsfc.nasa.gov/datasets/OMAERO_003/summary, and https://aeronet.gsfc.nasa.gov/ respectively.
